# The Economic Burden Associated with the Management of Different Stages of Breast Cancer: A Retrospective Cost of Illness Analysis in Saudi Arabia

**DOI:** 10.3390/healthcare9070907

**Published:** 2021-07-18

**Authors:** Ahmed Alghamdi, Bander Balkhi, Shahad Alqahtani, Hamoud Almotairi

**Affiliations:** 1 Department of Clinical Pharmacy, College of Pharmacy, King Saud University, Riyadh 11451, Saudi Arabia; bbalkhi@ksu.edu.sa (B.B.); halmotairi@ksu.edu.sa (H.A.); 2 Pharmacy Department, King Fahad Medical City, Riyadh 11451, Saudi Arabia; ssmalqahtani@kfmc.med.sa

**Keywords:** breast cancer, economic burden, cost of illness

## Abstract

Globally, breast cancer management is associated with a heavy economic burden, but its impact in Saudi Arabia has not been fully quantified. The aim of this study was to estimate the economic burden of breast cancer management at various disease stages, in Saudi Arabia, from a payer perspective. We conducted a retrospective, multicenter cost of illness study in two governmental healthcare centers from January to December 2018, using the data of 300 patients at different breast cancer stages. A micro-costing, bottom-up method was used, and descriptive and inferential statistics were analyzed. The total estimated cost for treating breast cancer during the study period was $13.345 million USD, with the average cost per patient ranging from $14,249 USD in stage I to $81,489 USD in stage IV (*p* < 0.001). Medication cost was the main driver of total healthcare spending, followed by hospitalization and diagnostic tests. The cost of targeted therapy drugs represented 67% of the total medication costs, mostly driven by trastuzumab-based regimens. The economic burden of breast cancer management in Saudi Arabia is substantial and increases significantly with disease advancement. Early detection screening programs, evaluating the value of highly expensive interventions, and considering biosimilars, may contribute toward cost savings.

## 1. Introduction

Breast cancer is the most common type of cancer among females worldwide, accounting for one in every four diagnosed cancer cases; in 2018 alone, more than 2.1 million cases were diagnosed [1]. Breast cancer is also considered the leading cause of mortality among females, with 181,004 deaths reported globally in 2017 [2]. In Saudi Arabia, breast cancer is the predominant type of cancer with an overall incidence rate of 14.8% among both genders, accounting for 29.7% of cancer among females. Moreover, the breast cancer mortality rate in Saudi Arabia was estimated at 8.5% of all cancer-related deaths [3].

Breast cancer is considered a curable disease due to the development of advanced treatment options, which include targeted therapy, and the adoption of early detection and screening programs. These advances have resulted in a substantial improvement in breast cancer management and patient quality of life. Additionally, breast cancer has become increasingly survivable, especially when detected and treated in its early stages [4]. It has been reported that the survival rate of patients diagnosed at an early stage (I and II) may reach 85–95%, compared to 30–70% for those diagnosed at an advanced stage (III and IV) [5,6,7].

Despite substantial progress with regard to breast cancer management in recent years, incidence and mortality rates are still increasing in both developed and developing countries. This has led to significant increases in global healthcare costs related to patient care, in addition to the challenges faced in allocating sufficient healthcare resources to breast cancer patients, during the different phases of care for the disease [8,9].

It is well documented that breast cancer imposes a significant economic burden on healthcare systems and societies, especially during the first year following diagnosis. However, breast cancer also has a substantial long-term economic impact, as breast cancer survivors require continuous inpatient and outpatient care [9,10,11].

The economic burden of breast cancer management has been estimated in many countries. For example, in the United States (US), the total cost of breast cancer management reached $16.5 billion US Dollar (USD) in 2010, increasing to $20.5 billion USD in 2020 [12]. In other countries, such as South Korea and Spain, the estimated cost of breast cancer was $940.75 million USD and €518 million Euro (EUR), respectively [13,14]. The breast cancer stage at diagnosis is considered a major predictor of resource utilization and cost, with patients at advanced stages requiring more intensive treatment. This is associated with significantly higher consumption of healthcare resources and, consequently, higher costs compared to those at early stages of the disease [15].

A recent systematic review reported that the mean treatment cost for a breast cancer patient was estimated at $29,724 USD at stage I, $39,322 USD at stage II, $57,827 USD at stage III, and $62,108 USD at stage IV, with therapeutic drug costs serving as the major cost driver, especially in advanced disease. Thus, estimating the cost per patient at each breast cancer stage is important for evaluating the economic burden of breast cancer, as well as guiding healthcare decisions toward the effective allocation of healthcare resources [16]. In Saudi Arabia, the number of breast cancer cases was estimated at 3629 in 2018, and it is projected to reach 6886 by 2040. Furthermore, it has been reported that the majority of breast cancer patients in Saudi Arabia are diagnosed at advanced stages, which may substantially increase resource utilization and spending [17,18]. Although breast cancer imposes a heavy economic burden on healthcare systems and societies in many countries, its economic impact has not been fully studied in Saudi Arabia. The purpose of this study was to improve the understanding of the cost distribution at different stages of breast cancer treatment, using real-world data, and to explore the factors related to cost increases among Saudi patients. The results of this study may assist healthcare policymakers to better estimate the costs attributed to breast cancer at its various stages, as well as improve policies for the efficient allocation of limited healthcare resources and spending control.

## 2. Materials and Methods

### 2.1. Design and Population 

We conducted a retrospective cross-sectional, prevalence-based, multicenter cost of illness (COI) study, at King Fahad Medical City (KFMC) and King Saud University Medical City (KSUMC) oncology centers in Riyadh, Saudi Arabia. Using electronic medical records (EMRs), all patients diagnosed with breast cancer aged 18 years and above based on the International Statistical Classification of Diseases and Related Health Problems (ICD-10) were included. The study was conducted from a payer perspective to include estimations of direct medical costs over 1 year between January and December 2018. To estimate the economic burden and amount of resources consumed, we developed a COI model using a micro-costing, bottom-up approach to estimate the cost of healthcare resources directly related to the treatment of breast cancer for each patient on the basis of the cost of individual units of service performed.

### 2.2. Data Collection 

We collected the demographics and disease characteristics of patients diagnosed with breast cancer, using EMRs. Data included age, weight, body mass index (BMI), menopausal status, history of cancer, marital status, comorbidities, breast cancer stage, medications, and therapeutic regimens. In line with the payer perspective of our study, we only estimated healthcare resource costs that were directly related to breast cancer management, including the costs of hospitalization, procedures, medications, radiotherapy, diagnostic and lab tests, and outpatient visits. Costing and pricing data were obtained from the KFMC and KSUMC business centers.

For cost estimation, we measured and quantified healthcare resources used for, or interventions performed on, each patient during the period of study, before estimating the unit costs for such interventions. Accordingly, we calculated the total direct cost as the product of the unit cost and the quantity consumed, and we estimated the overall direct cost per patient, per disease stage. To reduce the discrepancy between estimated costs and enhance the generalizability of the results, weighted averages of both institutional cost types were calculated. All cost calculations were adjusted for inflation to the year 2021 and are presented in USD (1 USD = 3.75 Saudi Riyal (SAR)), and no discounting was applied as the study was limited to a 1 year period.

### 2.3. Statistical Analysis

We performed descriptive statistics, including the calculation of means, standard deviation (SD), and frequencies as appropriate to patient demographics and cost categories. Additionally, we used inferential statistics—including analysis of variance (ANOVA)—as needed, to study the differences between different stages. All *p*-values below 0.05 were considered statistically significant. We conducted statistical analyses using the Statistical Package for the Social Sciences (SPSS) V21.0 (IBM Corp., Armonk, NY, USA).

## 3. Results

The study included 300 patients who were diagnosed with breast cancer during 2018. Table 1 provides a summary of patient demographics and disease characteristics. The mean age (SD) of the patients was 48 (10) years, with approximately 67.3% of patients falling into the age category of 40–60 years. The mean BMI (SD) was 29.2 (6.7) with nearly 40.7% of patients classified as obese. In total, 69.3% of patients were married, and approximately 76.0% were of premenopausal status. Most patients (88.0%) had a personal history of recurrent breast cancer, while only 13.0% had a family history of breast cancer. Patients diagnosed with stage IV breast cancer represented 32.0% of the study population, whereas 30.0% were at stage III, 24.7% were at stage II, and 13.3% were at stage I. 

**Figure 1:**
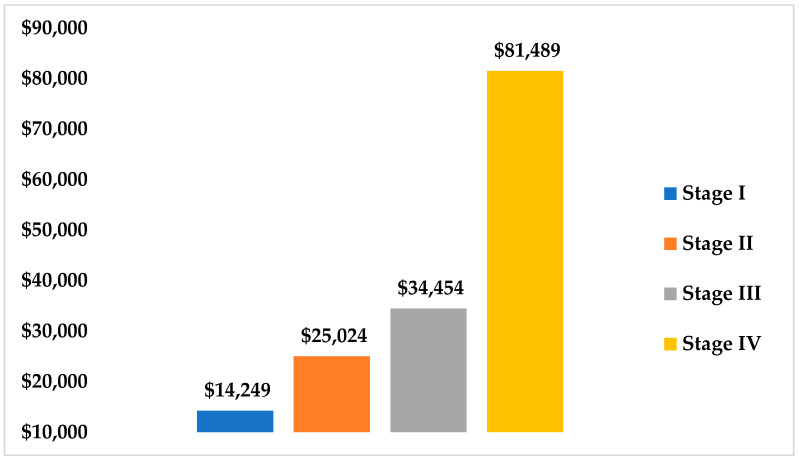
Estimated annual cost of breast cancer management (in USD), per disease stage.

The total direct medical cost of breast cancer management across all stages, during the study period, amounted to $13,345,526 USD. The total spending on patients with stage IV breast cancer was $7,822,911 USD, compared to $3,100,886 USD; $1,851,775 USD; and $569,953 USD for treatment of those with stage III, stage II, and stage I disease, respectively. As shown in Figure 1, the average annual cost per patient varied with breast cancer stage, ranging from $14,249 USD per patient with stage I to $81,489 USD per patient battling stage IV (*p* < 0.001).

Table 2 provides a summary of the average cost per patient, for each healthcare resource utilized during the study period. The annual direct medical cost related to the use of medication was estimated at $4778 USD for stage I disease, compared to $14,361 USD, $21,483 USD, and $65,518 USD for treatment of stage II, stage III, and stage IV breast cancer, respectively. Moreover, the average annual hospitalization costs were highest for patients with stage III disease, followed by those for stage IV cancer patients, at $4601 USD and $4081 USD, respectively. Furthermore, the average annual direct procedure-related costs ranged from $2867 USD to $3718 USD in the treatment of patients with stage I–III disease, decreasing to $2817 USD for patients with stage IV cancer. Average direct costs related to diagnostic and laboratory tests were estimated at $2056 USD and $2180 USD for stage I and IV disease, respectively, decreasing to $1789 USD for stage II and $1733 USD for stage III disease. 

**Figure 2:**
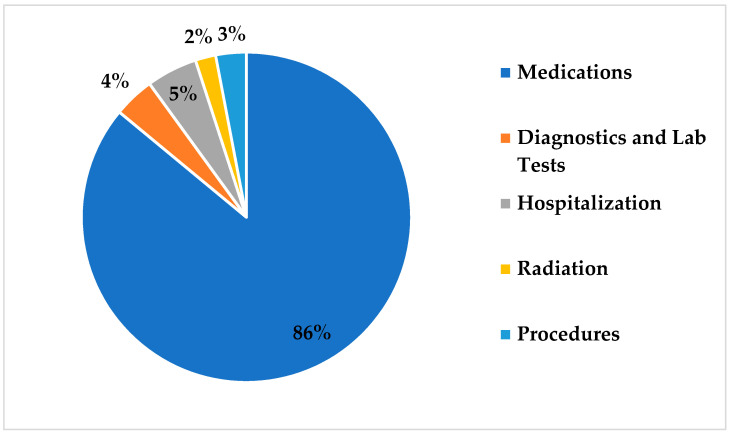
Major drivers of direct medical costs in breast cancer management.

**Figure 3:**
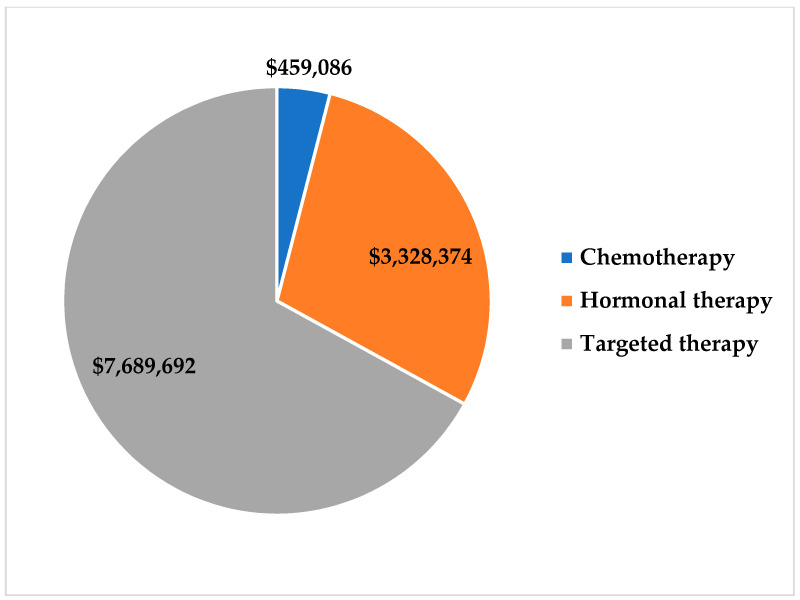
The estimated annual cost for drug categories used in breast cancer treatment (in USD).

Figure 2 illustrates the major drivers of total spending across all breast cancer stages. Hospital admission and outpatient visits accounted for 5% of total spending, while diagnostic tests, procedures, and radiotherapy accounted for 4%, 3%, and 2%, respectively. This study found that approximately 86% of total costs—$11,477,152 USD in total—were related to the use of medications. Of this, about 73% was spent on treatment of patients with stage IV cancer, followed by 16%, 9%, and 2%, respectively, in descending order of disease stage. Approximately 67% of total spending on medication was associated with targeted therapy drugs, primarily (94%) comprising trastuzumab, either alone or in combination with pertuzumab (Figure 3). Hormonal therapy accounted for 29% of total spend on medications and was mainly (65%) driven by the administration of fulvestrant and letrozole. The use of chemotherapy drugs accounted for 4% of the total spend on medications.

## 4. Discussion

This study provided a clear indication of the distribution of healthcare resources and costs during the management of different stages of breast cancer, in Saudi Arabia. It also offered first insights into the total direct costs associated with the management of breast cancer, based on real-world data from Saudi patients, which are valuable in making decisions regarding treatment. Herein, the economic burden of breast cancer management was proven to be substantial. The estimated total direct cost of managing patients with breast cancer during the study period and across all stages was $13,345,526 USD, an extremely high amount, considering that only 300 patients were included in the study.

Our findings demonstrated that the direct costs were variable and increased significantly as the disease progressed to advanced stages. Comparatively, the average annual cost per patient was higher during advanced stages of the disease, at factors of 1.8, 2.4, and 5.7 times the cost per patient with stage I disease, in stages II, III, and IV, respectively. This could mainly be attributed to the increased need for treatment and higher consumption of healthcare resources by patients with advanced stages of disease, especially stages III and IV [11].

Our estimates are aligned with the outcomes of several studies conducted in other countries including the US, Canada, Belgium, the United Kingdom (UK), Italy, and Jordan, which showed a strong correlation between disease stage and total cost of treatment; patients with more advanced disease require more intensive treatment and use more healthcare resources than patients with early-stage disease [9,10,19,20,21]. The availability of costing data per cancer stage is crucial for assessing the value of potential breast cancer treatment and prevention programs. It has been documented that breast cancer is mainly diagnosed at an advanced stage in Saudi Arabia, and early screening is limited. Hence, our study confirmed the importance of increasing public and healthcare awareness, establishing prevention clinics, and improving early breast cancer detection programs, as major health policy initiatives to minimize the costs of managing more advanced stages of the disease [18,22,23,24]. Evidence has shown that early diagnosis and consequent treatment could improve savings by up to 11.35% of the total cost [23]. Future studies may focus on measuring the cost of implementing a national screening program for early breast cancer detection and diagnosis, as well as evaluating the cost and benefit of such programs in Saudi Arabia.

Our study found that medication costs were the main driver of total cost, accounting for 86.0% of total healthcare spending on breast cancer. Furthermore, we found that medication costs increased substantially with advancing cancer stages, representing 33.5%, 57.4%, 62.4%, and 80.4% of the cost at stages I, II, III, and IV, respectively. These findings were similar to those of previously reported studies across different countries, in which medication costs were identified as the main cost drivers for all disease stages [24,25,26].

We further found that targeted therapy drugs, which are very expensive, especially those related to trastuzumab regimens, were significant drivers of cost, accounting for 67.0% of the total breast cancer medication spend in this study. This supports the outcomes of a previous study that identified targeted therapy drugs as major contributors to medication-related spending on breast cancer patients in Saudi Arabia [27]. This is also the case in other Middle East and North Africa (MENA) countries such as Jordan, where the total cost of trastuzumab accounted for almost 80.0% of medication costs evaluated in one study, and Morocco, where one course of trastuzumab can amount to as much as 60 times the cost of certain other chemotherapy drugs [25,28]. In the US, the use of targeted therapies has reportedly steadily increased over the last 10 years, with major economic impact, mainly attributable to higher drug prices [29].

The findings of our study highlighted the serious need for adopting approaches to lower the cost of targeted therapy drugs, as these are the major drivers of breast cancer costs, especially at advanced disease stages. With the increasing prevalence of breast cancer in Saudi Arabia and the large number of patients who require targeted therapy, it is crucial to improve access to these effective medications. Moreover, approaches to lowering the costs of these medications should also be improved, considering the typically limited healthcare budgets available to fund them. One such approach that may lead to substantially decreased cost of targeted therapy is switching to biosimilar forms of these drugs that provide good cost-saving opportunities, considering their comparable efficacy at a much lower price [30].

It has been estimated that substitution of both intravenous (IV) and subcutaneous trastuzumab with IV biosimilar trastuzumab in Saudi Arabia could amount to cost savings of roughly $30 million USD [31]. Moreover, several European countries have made similar observations, suggesting that a switch to biosimilar trastuzumab could result in savings of up to €2.27 billion EUR, over 5 years [32]. Another approach is to evaluate the effectiveness of medications used in the different treatment options, including targeted therapy drugs, before using them in hospitals or national formularies. Thus, the application of pharmacoeconomic principles such as cost effectiveness and budget impact analyses would be beneficial in assessing the value and affordability of these expensive medications, as well as help in optimizing the use of limited healthcare resources [33]. Furthermore, this could be translated into polices including price discounts, thus improving payment models, in addition to adapting a value-based healthcare approach linking patient-reported outcomes to payment and reimbursement. This will be critical for the future sustainability of the health system, thereby improving quality of care while maintaining or reducing the cost [34].

Our study also showed that hospitalization costs, albeit consistently lower than medication costs, increased as the disease progressed. This finding is, however, inconsistent with other studies that reported higher hospitalization costs for breast cancer patients; this may be ascribed to differences in the healthcare systems, patient populations, and treatment protocols evaluated [35,36]. Assessing the impact of decreased hospitalization costs on overall cost and quality of life, compared to that reported in other countries, would be an interesting topic for future investigation.

As our study was conducted from the payer’s perspective, considering only direct medical costs, we did not account for the impact of other types of costs, such as direct nonmedical and indirect costs. In countries such as Korea and Sweden, cancer management costs are largely driven by indirect costs. Additionally, the total direct nonmedical costs are estimated to reach over $50.69 million USD, mostly driven by caregiver costs [13,36]. Future studies should, therefore, consider the impact of both indirect and direct nonmedical costs on the overall economic burden of breast cancer management in Saudi Arabia.

## 5. Conclusions

Breast cancer is considered a major health problem in Saudi Arabia, and its management imposes a heavy burden on the economy, which significantly increases with advanced disease stages. The cost of highly expensive targeted therapy drugs is a major driver of total direct medical costs, especially in the treatment of disease stages III and IV. Our findings highlight the importance of early breast cancer detection and screening programs as tools to help lower costs and increase savings. Furthermore, healthcare decisionmakers should consider the use of approved biosimilars for targeted therapy, as these drugs provide comparable therapeutic efficacy at lower prices, potentiating cost savings. The findings of our study may guide future policies and research toward evaluating the added value and the potential affordability of new and expensive breast cancer therapies and preventive programs, to allocate resources more efficiently, especially for patients at advanced stages of the disease.

## Figures and Tables

**Table 1:** Patient demographics and disease characteristics.

Variable	Count	Frequency (%)
Mean age (SD) = 48 (10)		
Below 40	65	21.7%
40–60	202	67.3%
Above 60	33	11.0%
Mean BMI (SD) = 29.2 (6.7)		
Underweight	18	6.0%
Normal	76	25.3%
Overweight	84	28.0%
Obese	124	40.7%
Marital status		
Single	75	25.0%
Married	208	69.3%
Divorced/widowed	17	5.7%
Menopausal status		
Premenopausal	228	76.0%
Postmenopausal	72	24.0%
Family history of breast cancer		
Yes	39	13.0%
No	261	87.0%
Personal history of breast cancer		
Yes	264	88.0%
No	36	12.0%
Breast cancer stage		
I	40	13.3%
II	74	24.7%
III	90	30.0%
IV	96	32.0%

Abbreviations: BMI, body mass index; SD, standard deviation.

**Table 2:** The estimated average annual direct medical cost per patient (in USD), at each breast cancer stage.

	Breast Cancer Stages			
Cost Category	I	II	III	IV
Medication	$4778 ($1837)	$14,361 ($3130)	$21,483 ($4279)	$65,518 ($9362)
Diagnostic tests	$2056 ($230)	$1789 ($220)	$1733 ($271)	$2180 ($354)
Radiation	$2871 ($24)	$2890 ($33)	$2919 ($50)	$2893 ($74)
Hospitalization	$1677 ($500)	$2604 ($747)	$4601 ($1264)	$ 4081 ($1089)
Procedures	$2867 ($1125)	$3380 ($1647)	$3718 ($1117)	$ 2817 ($1601)

Note: Costs are presented as the mean (SD).

## Data Availability

Data sharing not applicable.
